# The Functional Role and Regulatory Mechanism of Bromodomain-Containing Protein 9 in Human Uterine Leiomyosarcoma

**DOI:** 10.3390/cells11142160

**Published:** 2022-07-10

**Authors:** Qiwei Yang, Maria Victoria Bariani, Ali Falahati, Azad Khosh, Ricardo R. Lastra, Hiba Siblini, Thomas G. Boyer, Ayman Al-Hendy

**Affiliations:** 1 Department of Obstetrics and Gynecology, University of Chicago, Chicago, IL 60637, USA; bariani@bsd.uchicago.edu (M.V.B.); hsiblini@bsd.uchicago.edu (H.S.); aalhendy@bsd.uchicago.edu (A.A.-H.); 2 Department of Biology, Yazd University, Yazd 8915818411, Iran; ali.falahati@yazd.ac.ir (A.F.); azadkhosh11@gmail.com (A.K.); 3 Department of Pathology, University of Chicago, Chicago, IL 60637, USA; ricardo.lastra@uchospitals.edu; 4 Department of Molecular Medicine, Institute of Biotechnology, University of Texas Health Science Center at San Antonio, San Antonio, TX 78229, USA; boyer@uthscsa.edu

**Keywords:** uterine leiomyosarcoma, bromodomain-containing protein 9, transcriptome analysis, epigenome, miRNAs, TP-472

## Abstract

Uterine leiomyosarcoma (uLMS) is the most common type of uterine sarcoma associated with poor prognosis, high rates of recurrence, and metastasis. There is currently limited information about uLMS molecular mechanisms of origin and development. Bromodomain (BRD)-containing proteins are involved in many biological processes, most notably epigenetic regulation of transcription, and BRD protein dysfunction has been linked to many diseases including tumorigenesis. However, the role of BRD proteins in the pathogenesis of uLMS is unknown. Here, we show for the first time that BRD9 is aberrantly overexpressed in uLMS tissues compared to adjacent myometrium. BRD9 expression is also upregulated in uLMS cell lines compared to benign uterine fibroid and myometrium cell lines. Inhibition of BRD9 using the specific inhibitor (TP-472) suppressed uLMS cell proliferation via inducing apoptosis and cell cycle arrest. To further characterize the mechanistic basis for TP-472 inhibition of uLMS cell growth, we performed a comparative RNA-seq analysis of vehicle-treated and TP-472-treated uLMS cells (*n* = 4 each). Bioinformatics analysis revealed that TP-472 treatment distinctly altered the uLMS cell transcriptome. Gene set enrichment analysis identified critical pathways altered by BRD9 inhibition, including interferon-alpha response, KRAS signaling, MYC targets, TNF-a signaling via NFkB, and MTORC1 signaling. Parsimonious gene correlation network analysis identified nine enriched modules, including cell cycle and apoptosis modules. Moreover, the ENCODE Histone Modifications gene set and TargetScan microRNA analysis in Enrichr suggested that TP-472-induced BRD9 inhibition may alter the uLMS cell transcriptome by reprograming the oncogenic epigenome and inducing miRNA-mediated gene regulation. Therefore, BRD9 constitutes a specific vulnerability in malignant uLMS, and targeting non-BET BRD proteins in uLMS may provide a promising and novel strategy for treating patients with this aggressive uterine cancer.

## 1. Introduction

Uterine leiomyosarcoma (uLMS) is a rare uterine cancer, representing 1–2% of all uterine malignancies [1]. The annual incidence of uLMS is approximately 6 per 1,000,000 women. The 5-year survival rate for all patients ranges between 25 and 76%, while survival for women with metastatic disease at the time of initial diagnosis approaches only 10–15% [2]. Irrespective of treatment, uLMS is characterized by poor prognosis [3], and many uLMS patients exhibit resistance to currently available therapies, as evidenced by high rates of both recurrence and progression [4,5,6].

A significant body of foundational data supports a key role in epigenetic dysregulation of gene expression in oncogenesis [7,8,9,10]. For example, altered epigenetic modifications, including DNA methylation and histone modifications, as well as aberrant expression of non-coding RNAs, have been variously shown to contribute to tumor initiation and development [9,11,12,13,14,15,16,17]. Notably, as the “readers” of lysine acetylation, bromodomain (BRD)-containing proteins are responsible for transducing regulatory signals carried by acetylated lysine residues into various biological phenotypes [18]. BRD proteins can exert a wide variety of functions via multiple gene regulatory mechanisms [19] and deregulation of BRDs is involved in many diseases, including cancer [20,21,22,23,24].

Bromodomain-containing protein 9 (BRD9) is a newly identified subunit of the non-canonical barrier-to-autointegration factor (ncBAF) complex and a member of the bromodomain family IV [25]. Studies have demonstrated that BRD9 plays an oncogenic role in multiple cancer types, by regulating tumor cell growth. The connection of BRD9 with PI3K pathway [26], miR RNAs [27], and STAT5 [28] is implicated in cancer progression. However, the role and mechanism of BRDs in the pathogenesis of uLMS are unknown. Accordingly, the present study aimed to determine whether and how BRD9 contributes to aberrant uLMS cell growth, with important implications for the development of novel treatment options for this highly aggressive uterine cancer.

## 2. Materials and Methods

### 2.1. Tissues and Immunohistochemistry

The uLMS tissues (*n* = 9) were obtained from the University of Chicago Tissue Bank. Approval from the Institutional Review Board (#20-1820) at the University of Chicago was obtained for the retrospective chart review of uLMS patients. Informed consent was obtained from all the patients participating in the study before surgery. The cases with an initial diagnosis of uLMS at University of Chicago Hospital were reviewed (Table 1), and the diagnosis was confirmed by H&E evaluation and immunohistochemistry, when required.

The mean age of uLMS patients was 54.0 ± 6.9 years, with a range of 42 to 61 years. The clinicopathological data about the patient cohort and their uLMS samples were summarized in Table 1. The original blocks were retrieved from the tissue bank and were sectioned onto coated slides at a thickness of 5 µM. One section was stained with hematoxylin and eosin to evaluate the morphology of each spot, and the remaining slides were used for IHC analysis. Briefly, sections were deparaffinized with xylene, and rehydrated by being passed through decreasing concentrations of ethanol in water. Then, antigen retrieval and quenching of endogenous peroxidases were performed. Sections were incubated with primary antibodies (HMB45, SMA, desmin, and BRD9) (Table 2) in a humidity chamber overnight at 4 °C and developed with peroxidase labeled-dextran polymer followed by diaminobenzidine (DAKO Envision Plus System; DAKO Corporation, Carpinteria, CA, USA). Sections were counterstained with Gill’s Hematoxylin (Fisher, Pittsburgh, PA, USA). To determine the percentage of BRD9 positive cells, the samples were analyzed using the positive cell detection command on QuPath software. Different thresholds were set to categorize cells according to nuclei staining intensity: negative, weak, moderate, and strong intensity. Smooth muscle was used a positive tissue for SMA and desmin. Melanoma was used a positive control for HMB45. Human testis was used as positive control for BRD9 IHC staining.

### 2.2. Cells and Reagents

The leiomyosarcoma (uLMS) cell line (SK-UT1, ATCC^®^ HTB-114^TM^) (ATCC, Manassas, VA, USA) was cultured and maintained in ATCC-formulated Eagle’s Minimum Essential Medium with 10% of fetal bovine serum. In addition, the uterine sarcoma cell line (MES-SA) (ATCC, Manassas, VA, USA) was cultured and maintained in McCoy’s 5A medium. The immortalized human leiomyoma cell line (HuLM) and immortalized human uterine smooth muscle (UTSM) cells were a generous gift from Professor Darlene Dixon. The HuLM and UTSM cell lines were cultured and maintained in phenol red-free, Dulbecco’s Modified Eagle Medium: Nutrient Mixture F-12. We used these cell lines covering the spectrum from normal cell line (UTSM), benign uterine tumor cell line (HuLM), and uterine malignant cell lines (uLMS) to better understand the tumor progression linking to the BRD9 dysregulation in uLMS. BRD9 inhibitor (iBRD9) TP-472 was purchased from Tocris (Cat# 6000, Minneapolis, MN, USA).

### 2.3. Proliferation Assay

Cell proliferation was measured using trypan blue exclusion assay; 4 × 10^4^ cells per well were seeded into 12-well tissue culture plates, treated with the iBRD9 (TP-472) at a dose range from 1–25 µM for 48 h. This assay was performed three times in triplicate.

### 2.4. RNA Extraction and Gene Expression

Total RNA was isolated using Trizol reagent (Invitrogen, Carlsbad, CA, USA). The concentration of total RNA was determined using NanoDrop (Thermo Scientific, Waltham, MA, USA). One microgram of total RNA from each sample was reverse transcribed to complementary DNA (cDNA) using the High-Capacity cDNA Transcription Kit (Thermo Scientific, Waltham, MA, USA). Quantitative real-time polymerase chain reaction (qRT-PCR) was performed to determine the messenger RNA (mRNA) expression of several genes listed with their primer sequences in Table 2. The real-time PCR reactions were performed using CFX96 PCR instrument using SYBR Green Supermix (Bio-Rad, Hercules, CA, USA). *18S* was used as an internal control. The results are presented as relative gene expression using CFX Maestro^TM^. The assay was performed three times in triplicate.

### 2.5. Protein Extraction and Western Blot

Cells were collected and lysed in RIPA lysis buffer with protease and phosphatase inhibitor cocktail (Thermo Scientific, Waltham, MA, USA), the protein was quantified using the Bradford method (Bio-Rad Protein Assay kit). The information about primary antibodies, including antibody dilution and source of antibodies is listed in Table 2. The antigen–antibody complex was detected with Trident Femto Western HRP substrate (GeneTex, Irvine, CA, USA). Specific protein bands were visualized using ChemiDoc XRS þ molecular imager (Bio-Rad, Hercules, CA, USA).

### 2.6. RNA-Sequencing

To determine the mechanism underlying the inhibitory effect of BRD9 inhibition on the uLMS, the SK-UT-1 cells were treated with iBRD9 TP-472 (5 µM) for 48 h. RNA was isolated using Trizol. RNA quality and quantity were assessed using the Agilent bio-analyzer. Strand-specific RNA-SEQ libraries were prepared using a TruSEQ total RNA-SEQ library protocol (Illumina provided). Library quality and quantity were assessed using the Agilent bio-analyzer and libraries were sequenced using an Illumina NovaSEQ6000 (illumine provided reagents and protocols).

### 2.7. Transcriptome Profiles Analysis

#### 2.7.1. Transcriptome Data Analysis

A variety of R packages was used for this analysis. All graphics and data wrangling were handled using the tidyverse suite of packages. All packages used are available from the Comprehensive R Archive Network (CRAN), Bioconductor.org, or Github. The reads were mapped to the human reference transcriptome using STAR, version 2.6.1d (GitHub, Inc., San Francisco, CA, USA). The quality of raw reads, as well as the results of STAR mapping, are generated using fastqc and multiqc. Raw reads were mapped to the human reference transcriptome using Salmon, version 1.4.0. After reading mapping with Salmon, (https://bioconductor.org/packages/release/bioc/html/tximport.html, accessed on 27 October 2021) was used to read Salmon outputs into the R environment. Annotation data from Gencode V34 was used to summarize data from transcript-level to gene-level.

#### 2.7.2. Integrated Bioinformatics Analysis

For Parsimonious gene correlation network analysis (PGCNA), normalized matrix of DEGs (Adjusted *p*-Values < 0.05 and −1.5 > fold-change > 1.5; *n* = 3583) and the top 80% of the most variance genes across samples (*n* = 10,848) were used to network construction. The matrix was used to calculate Spearman rank correlations for all gene pairs using the Python PGCNA2 package [29]. For each gene (row) in a correlation matrix, only the three most correlated edges per gene were retained. The correlation matrices were clustered 100 times using the fast unfolding of communities in a large network’s algorithm and the best (judged by modularity score) were used for downstream analysis. Gephi package with the ForceAtlas2 layout method was used to visualize the network [30]. Enrichment analyses were performed using the Enrichr web server (https://maayanlab.cloud/Enrichr/, accessed on 10 January 2022) through enrichR package in R software [31]. Top biological process GO terms with the false discovery rate (FDR) > 0.05 were analyzed using REVIGO to summarize and find a representative subset of the terms [32]. STRING database (https://string-db.org/, accessed on 27 April 2022), which is the online search tool to protein-protein network (PPI) construction, was used to reconstruct each modules network (A combined score ≥ 0.4 of PPI pairs was considered significant), then the Cytoscape software was employed to analyze the obtained networks. In addition, the Venn diagram online tool provided by VIB and Ghent University (http://bioinformatics.psb.ugent.be/webtools/Venn/, accessed on 27 April 2022) was used to investigate the intersection of DEGs and selected modules.

### 2.8. Quantitative Real-Time Polymerase Chain Reaction (qRT-PCR)

Quantitative real-time PCR was performed to determine the mRNA expression of genes as described previously [33]. Primers were purchased from Integrated DNA Technologies (IDT, Coralville, IA, USA) with primer sequences shown in Table 3. An equal amount of cDNA from each sample was added to the Master mix containing appropriate primer sets and SYBR green supermix (Bio-Rad) in a 20 µL reaction volume. All samples were analyzed in triplicates. Real-time PCR analyses were performed using a Bio-Rad CFX96. Cycling conditions included denaturation at 95 °C for 2 min followed by 40 cycles of 95 °C for 5 s and 60 °C for 30 s then 65 °C for 5 s. Synthesis of a DNA product of the expected size was confirmed by melting curve analysis. Further, 18S ribosomal RNA values (internal control) were used to normalize the expression data and normalized values were used to create data graphs. Negative control has been performed by running the reaction without cDNA from Integrated DNA Technologies (IDT, Coralville, IA, USA).

### 2.9. Statistical Analysis

All experiments were conducted with at least three biological replicates. Comparison of two groups was carried out using Student *t*-test for parametric distribution and Mann–Whitney test for nonparametric distribution. Comparison of multiple groups was carried out by analysis of variance (ANOVA) followed by a post-test using Tukey for parametric distribution and Kruskal–Wallis test followed by a post-test Dunns for nonparametric distribution, using GraphPad Prism 5 Software. Data were presented as mean ± standard error (SE). In figures, ns, *, **, and *** indicate, not significant, *p* < 0.05, <0.01, and <0.001 respectively.

## 3. Results

### 3.1. Pathological Examination

**Figure 1:**
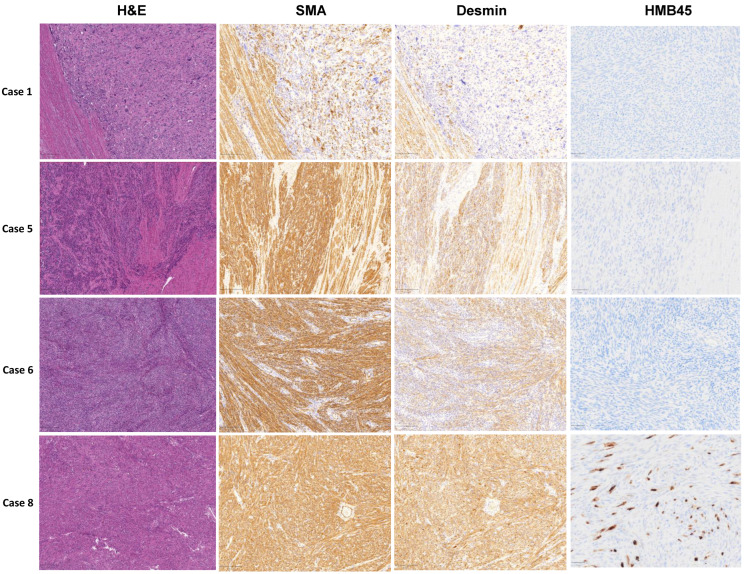
Morphological observation and IHC staining of SMA, desmin, and HMB45 in four representative human uLMS tissues and adjacent myometrium.

A total of nine patients with uLMS were identified. Of these, seven met the definition of conventional uLMS, while two met the definition of non-conventional uLMS resembling epithelioid uLMS and sex cord tumor. Patient information collected is shown in Table 1. The mean age of uLMS patients was 54.6 ± 7.4 years with a range of 42 to 62 years. Tumor size was 14.1 ± 7.9 cm in average (range: 2.9 cm to 27 cm). H&E staining exhibited a boundary between myometrium and uLMS (Figure 1). In cases with equivocal morphologic features, SMA, desmin, and HMB45 immunohistochemical stains were performed, confirming the diagnosis of uLMS. SMA and Desmin were all positive in 9/9 (100%) of cases by IHC analysis. For HMB45 IHC, seven and two of nine samples showed negative and positive HMB45 expression, respectively. The cases with HMB45 expression demonstrate only focal positivity and were considered insufficient to establish a diagnosis of PEComa (Figure 1, Table 1).

### 3.2. The BRD9 Expression Is Upregulated in uLMS Tissues Compared to Adjacent Myometrium from Women with uLMS

**Figure 2:**
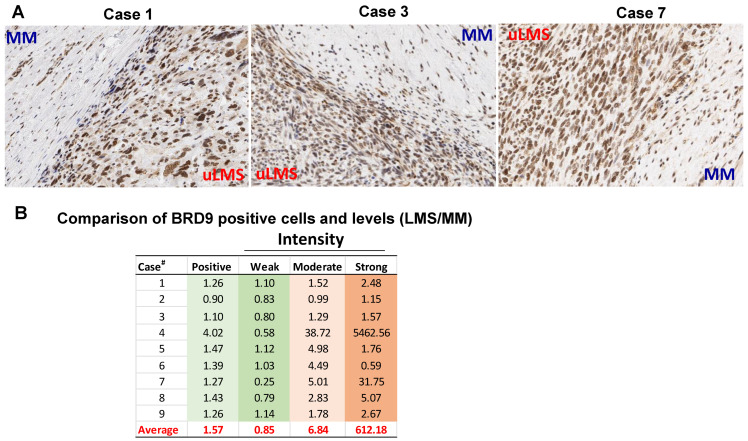
BRD9 expression is aberrantly upregulated in uterine uLMS compared to adjacent myometrium. (**A**) The expression of BRD9 in 3 representative uLMS tissues. (**B**) Quantitative comparison of BRD9 positive and expression levels between uLMS and myometrium in 9 uLMS cases.

To determine whether BRD9 is dysregulated, the comparison of BRD9 positive cells and expression levels was analyzed on uLMS tissues (*n* = 9) and adjacent myometrium (MM^+uLMS^) (*n* = 7). We observed that among seven cases (1–7) analyzed with both myometrium and uLMS, six cases exhibited an increased percentage of BRD9 positive cells in uLMS compared to MM^+uLMS^. Although the percentage of cells with a weak level of BRD9 expression is increased in only three out of seven uLMS cases compared to MM^+uLMS^ (Figure 2A,B), the percentage of cells with moderate and strong expression levels of BRD9 in uLMS is much higher than MM^+uLMS^ (Figure 2A,B). The uLMS tissues from cases #8–9 did not have matched myometrium; therefore, we compared the BRD9 expression of cases #8–9 with the average of BRD9 expression from cases #1–7 myometrium tissues. As shown in Figure 2B, the BRD9 expression in uLMS from cases #8–9 exhibited higher expression of BRD9 compared to the BRD9 expression in the myometrium. Among nine cases analyzed, eight out of nine cases exhibited an increase in BRD9 positive cells, moderate intensity, and strong intensity cells. The average fold changes of positive cells, weak intensity, moderate intensity and strong intensity in uLMS compared to MM^+uLMS^ are 1.57, 0.85, 6.84, and 612.18, respectively (Figure 2B).

### 3.3. BRD9 Levels Are Upregulated in uLMS Cell Lines

**Figure 3:**
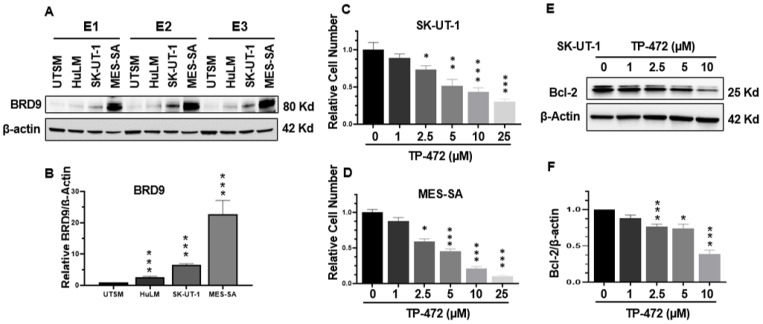
The protein levels of BRD9 in UTSM, HuLM, MES-SA, and SK-UT-1 cell lines and iBRD9 treatment. (**A**) The protein levels of BRD9 were measured by Western blot. E1, E2, and E3 represent three experiments (**B**) The protein levels of BRD9 were quantified using NIH Image J software. β-actin was used as an endogenous control. (**C**) Cell proliferation in SK-UT-1 cells in the presence or absence of TP-472; (**D**) Cell proliferation in MES-SA cells in the presence or absence of TP-472. (**E**) Bcl-2 levels in SK-UT-1 cells in the presence or absence of TP-472 measured by WB. (**F**) Quantitative analysis of Figure 3E using NIH Image J software. Three independent experiments were performed. * *p* < 0.05, ** *p* < 0.01, *** *p* < 0.001 compared between treated groups and control.

The constitutive (basal) expression levels of BRD9 in cell lines from human uterine smooth muscle (UTSM), human uterine leiomyoma (HuLM), and two different uLMS (SK-UT-1 and MES-SA) were evaluated by Western blot analysis. This analysis revealed that the expression levels of BRD9 exhibited a graded increase from normal and benign tumors to malignant uterine sarcoma cells. (Figure 3A,B).

### 3.4. Inhibition of BRD9 Decreased uLMS Cell Proliferation

TP-472 has been shown to inhibit BRD9 [34], therefore we selected TP-472 in our in vitro cell model to assess its effect on uLMS cell proliferation. Trypan blue exclusion assay was performed in SK-UT-1 and MES-SA cell lines treated for 48 h with TP-472 at dose ranges from 1–25 µM. The prolonged treatment (48 h) with iBRD9 (TP-472) showed a dose-dependent inhibitory effect on proliferation of both SK-UT-1 and MES-SA cells (Figure 3C,D). To determine if TP-472 treatment suppressed uLMS cell proliferation via apoptosis, the levels of BCL-2, which promotes cellular survival and inhibits the actions of pro-apoptotic proteins, were measured. As show in Figure 3E,F, TP-472 treatment significantly decreased the protein levels of BCL-2 in SK-UT-1 cells in a dose-dependent manner.

### 3.5. BRD9 Inhibition Causes Extensive Changes in the uLMS Cell Transcriptome

**Figure 4:**
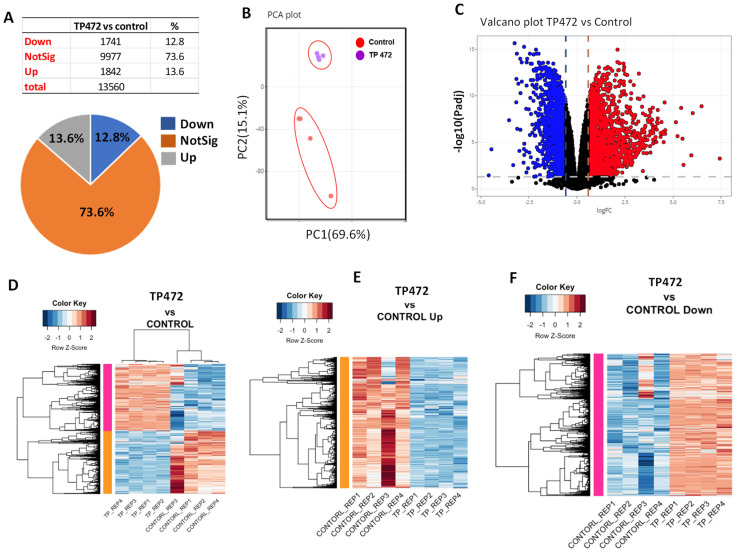
BRD9 inhibition causes extensive changes in the transcriptome of uLMS cells. (**A**) Pie chart showing the percentage of genes that exhibited changes in RNA expression between TP-472 and vehicle treatment groups as determined by RNA-seq. The cutoff value is 1.5-fold with an FDA < 0.05. (**B**) PCA plot of BRD9/inhibitor (TP-472) and vehicle control (DMSO), (**C**): Volcano plot showing the distribution of the DEGs between TP-472 and vehicle-treated SK-UT-1 cell line. The dashed line indicates the *p*-value significance threshold. (**D**) Heat map. Pearson correlation was used to cluster DEG (TP-472 vs. Control), which were then represented as a heatmap with the data scaled by Z score for each row. (**E**) Heat map. Pearson correlation was used to cluster DEG (TP-472 vs. Control, up genes), which were then represented as a heatmap with the data scaled by Z score for each row. (**F**) Heat map. Pearson correlation was used to cluster DEG (TP-472 vs. Control, down genes), which were then represented as a heatmap with the data scaled by Z score for each row.

To determine the mechanistic action of TP-472 inhibition on uLMS cells, RNA-sequencing analysis was performed in control and TP-472 treated cells (*n* = 4 each). As shown in Figure 4A, TP-472 treatment yielded 3583 differentially expressed genes (DEGs) (1741 down, 1842 up). Principal component analysis (PCA) demonstrated that the expression pattern of the TP-472 group clustered separately from the control group (Figure 4B). Volcano plot analysis revealed the distribution of pronounced changes in response to TP-472 treatment (Figure 4C). Heatmap analysis further demonstrated a distinguished expression pattern in SK-UT-1 cells treated with TP-472 as compared to the control group (Figure 4D–F).

#### 3.5.1. Pathway Analysis of DEGs upon TP-472 Treatment

**Figure 5:**
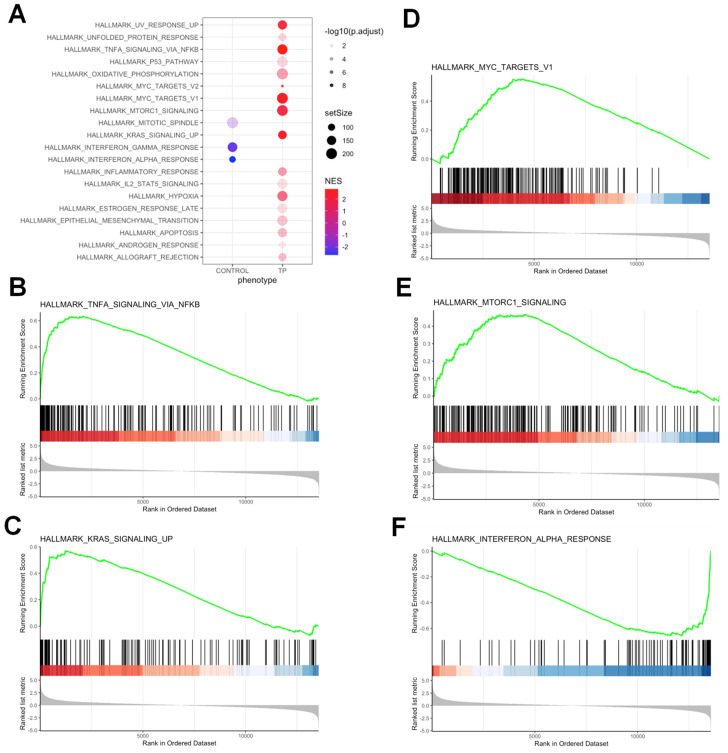
Hallmark analysis demonstrated the alteration of multiple pathways in SK-UT-1 cells in response to TP-472 treatment. (**A**) Functional pathways analysis identified significantly altered pathways in SK-UT-1 cells treated with TP-472. Significantly enriched gene sets (TP-472 vs. control) from GSEA using Hallmark biological processes in MSigDB. Gene count and significance levels are shown by the size and color of each circle, respectively. Pathways analysis revealed that several gene sets associated with TNF-a signaling via NFkB (**B**), KRAS signaling (**C**), MYC targets (**D**), MTORC1 signaling (**E**), and interferon-alpha response (**F**) were altered.

**Figure 6:**
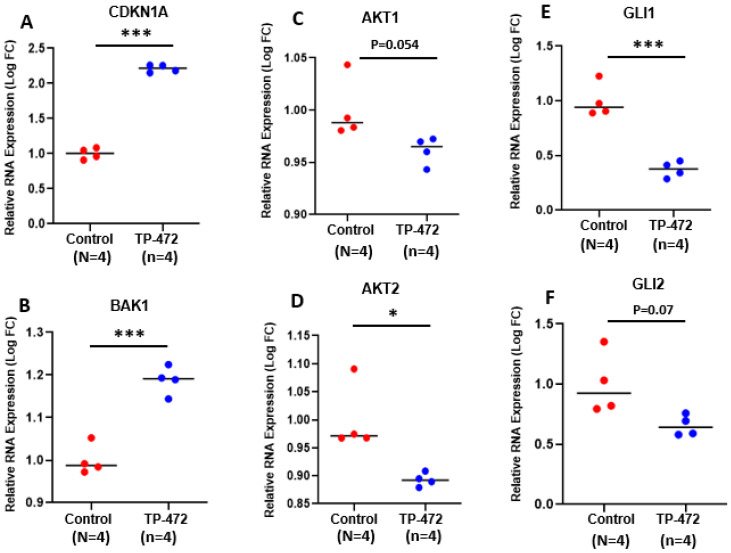
TP-472 induces altered expression of genes related to cell cycle, apoptosis, AKTs and Hedgehog pathways. (**A**) CDKN1A (**B**) BAK1 (**C**) AKT1 (**D**) AKT2 (**E**) GLI1 (**F**) GLI2. * *p* < 0.05; *** *p* < 0.001.

To gain insight into the biological changes induced by BRD9 inhibition globally, we performed Hallmark pathway analysis and identified several pathways that were significantly altered, including interferon-alpha response, KRAS signaling, MYC targets, MTORC1 signaling, and TNF-a response (Figure 5A–E). Figure 6 shows the expression of genes-related cell cycle and proliferation between control and TP-472-treated uLMS cells. Abnormally increased cell proliferation is one of the most notable characteristics of uLMS. P21 encoded by *CDKN1A* is a potent inhibitor of cell progression. As shown in Figure 6, *CDKN1A* expression is significantly higher in TP-472-treated uLMS cells compared to the control. In addition to cell cycle-related genes, the mRNA levels of BAK, the apoptosis regulator, were significantly increased in TP-472-treated SK-UT-1 cells compared to the control. AKT is at the crossroads of cell death and survival, playing a pivotal role in multiple interconnected cellular signaling mechanisms implicated in cell apoptosis and growth [35,36]. As shown in Figure 6, the expression of AKT was markedly decreased in TP-472-treated uLMS cells. The expression of AKT2 is significantly decreased in TP-472-treated uLMS cells (Figure 6). Previously we demonstrated that the Hedgehog pathway is activated in uLMS. In this study, we observed that TP-472 significantly decreased the expression of GLI1 and markedly reduced the GLI2 expression in uLMS cells. These data suggested that TP-472 induced cell cycle arrest and apoptosis and inhibited Hedgehog pathway in uLMS cells.

We also validated the expression of representative cell cycle- and apoptosis-related genes (CDKN1A and BAK) by q-PCR. As shown in Figure S1, the significant increase in expression of *CDKN1A* and *BAK1* upon iBRD9 treatment was consistent with RNA-seq data.

#### 3.5.2. Parsimonious Gene Correlation Network Analysis (PGCNA)

**Figure 7:**
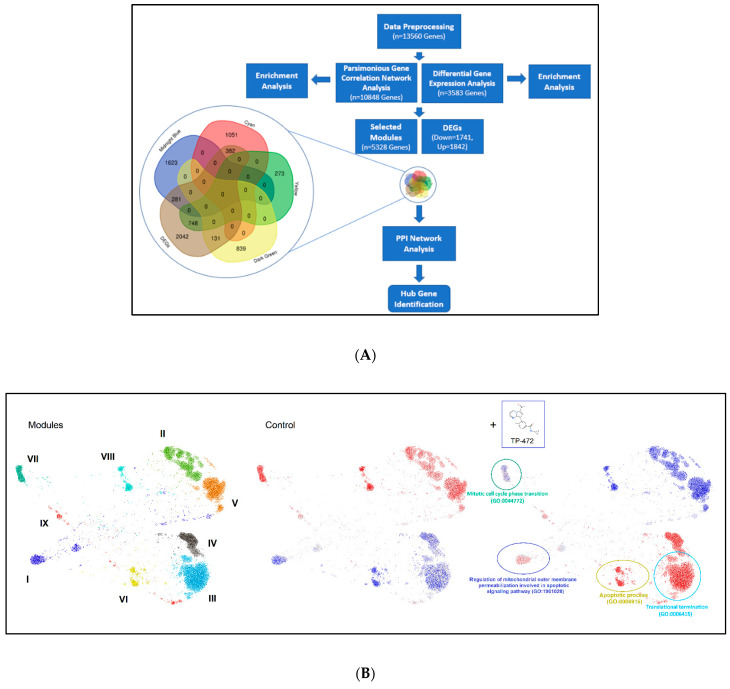
Network representation of the modular pattern of gene expression during the transition of control to TP-472 treatment. (**A**) Flowchart of modular pattern analysis, (**B**) Left: Nine network modules are color coded. Middle and right: Overlay of gene expression z-scores for all genes in the control and TP-472 conditions shown in blue (low) to red (high) z-score color scale. Four constructed modules, including mitotic cell cycle phase (GO: 0044772), regulation of mitochondrial outer membrane permeabilization involved in apoptotic signaling pathway (GO:1901028), apoptotic process (GO: 9996915), and translational termination (GO: 0006415) were enriched and highlighted. Note: Greek numerals are the index for Modules.

To identify a biologically meaningful gene module, we performed PGCNA. The pipeline of this study is shown in Figure 7A. The co-expression network was constructed using the PGCNA2 package in Python software from the annotated genes, in which nine modules were identified (Figure 7B, left panel). The enrichment results showed that the midnight blue and yellow modules were positively related to apoptosis; cyan and dark green modules were associated with the cell cycle (Figure 7B, middle and right panel). Therefore, subsequent enrichment analyses were performed to obtain further biological insight for the genes in the four constructed modules. The results for all four modules are shown in Table 4. From PGCNA analysis, apoptosis and cell cycle pathways were enriched upon TP-472 treatment. The top 40 hub genes (top 10 hub genes in each module) were screened out from the intersection of the Venn diagram between DEGs and four selected modules. The results are shown in Table S1. The intramodular connectivity of genes in the corresponding modules of interest was measured using the total number of edges linked to each gene, which is represented as the degree in Table S1. Notably, several Hub DEGs, including *MYC*, *FOS*, *JUN*, *EGR1*, *SHH* (sonic hedgehog pathway), *GRIN1*, *SOX9*, *HRAS, IMP3*, *CDK*, *TOP2A*, *TOP2B*, among others, are identified upon TP-472 treatment (Table S1).

#### 3.5.3. Inhibition of BRD9 Altered the Gene Expression Correlating to Histone Modifications

**Figure 8:**
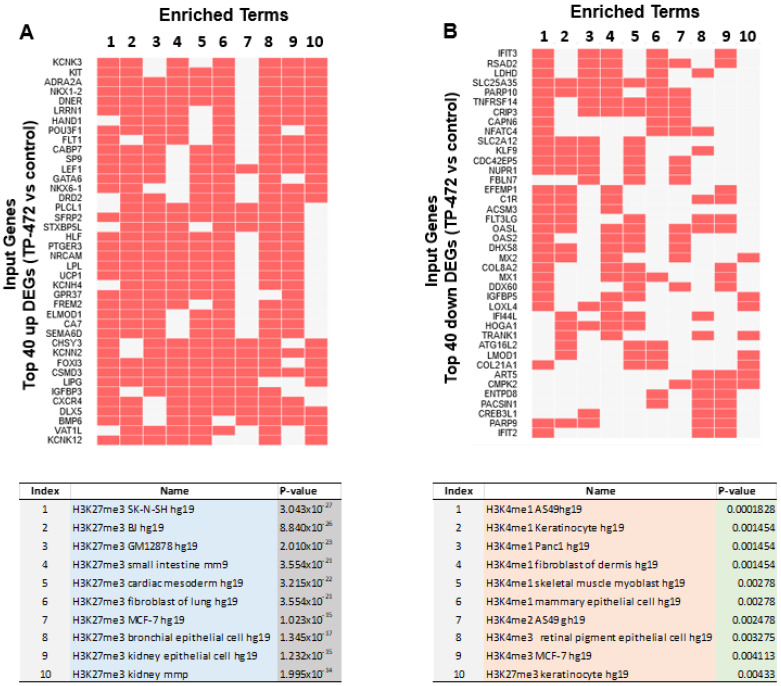
Visualization of the top 200 DEGs involved in histone modifications. (**A**) top panel: relation between histone modifications and top 40 DEGs among top 200 DEGs suppressed by TP-472 treatment (down). Lower panel: Top 10 altered epigenetic terms from top 200 down DEGs upon TP-472 treatment, (**B**) Top panel: the relation between histone modifications and top 40 DEGs among top 200 DEGs induced by TP-472 treatment (up). Lower panel: top 10 altered epigenetic terms from top 200 up DEGs upon TP-472 treatment. Note: Arabic numerals are the index for histone modifications.

To investigate whether TP-472 treatment led to transcriptional changes via epigenomic effects in uLMS cells, we performed enrichment analysis of epigenetic histone markers using the Enrichr web server. As shown in Figure 8A,B, DEGs between control and TP-472 were correlated with histone modifications. Notably, DEGs induced by TP-472 treatment were significantly linked to the H4K27me3, while those suppressed by TP-472 treatment were preferentially associated with H3K4me1, H3K4me2, H3K4me3, and H3K27me3 according to the ENCODE Histone Modifications gene set library. The top 10 terms are shown in the lower panel. These studies suggest that TP-472 treatment may alter the uLMS cell transcriptome via epigenetic mechanisms.

#### 3.5.4. Inhibition of BRD9 Altered Gene Expression Correlating to miRNA Regulation

**Figure 9:**
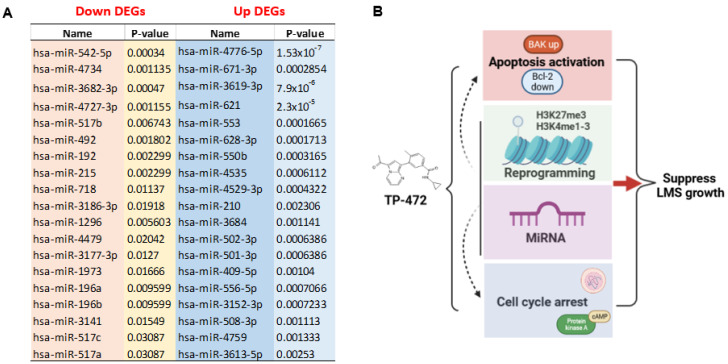
Visualization of the top 200 DEGs involved in miRNA regulation. (**A**) The relation between top 20 down/up miRNAs and top 200 up/down DEGs, (**B**) Model showing that TP-472 treatment activates apoptosis, induces cell cycle arrest, induces miRNA-mediated gene regulation, and reprograms pro-oncogenic epigenome in uLMS cells. Note: Arabic numerals are the index for histone modifications. Figure 9B was created using BioRender software.

We used TargetScan microRNA analysis in Enrichr to determine the mechanism underlying the regulation of DEG via miRNA in response to TP-472 treatment. As shown in Figure 9A, genes induced by TP-472 treatment (up) correlated with miRNAs, including hsa-miR-4776-5p, hsa-miR-671-3p, hsa-miR-3619-3p, hsa-miR-621, hsa-miR-553, among others. By contrast, genes suppressed by TP-472 treatment (down) correlated with a distinct set of miRNAs, including hsa-miR-542-5p, hsa-miR-4734, hsa-miR-3682-3p, and hsa-miR-4727-3p among others, suggesting that miRNAs may be involved in regulating gene expression in response to TP-472 treatment.

## 4. Discussion

Understanding the relationship between the epigenetic regulators and tumorigenesis is crucial for the manipulation of chromatin regulation in cancer therapy [37,38,39,40]. The present study has revealed that BRD9, as the readers of lysine acetylation for regulating the protein-histone association and chromatin remodeling, were upregulated in uLMS tissues and cells. Inhibition of BRD9 increased cell death, induced cell cycle arrest, altered several other critical biological pathways, and reprogrammed the onco-epigenome in uLMS cells, suggesting the critical role of BRD9 in the pathogenesis of uLMS.

The association and functional studies on BRDs in cancer biology have been extensively investigated in several types of cancer. For example, BRD specifies the control mixed lineage leukemia phenotype [41]. BRDs played a role in regulating key genes from chromatin and were associated with MLL fusion oncoproteins in leukemogenesis [42,43]. BRD2 is essential for proinflammatory cytokine production in macrophages. BRD2 and BRD4 physically associate with the promoters of inflammatory cytokine genes in macrophages. BRD inhibition by JQ1 can block this association and reduce the IL-6 and TNF-a levels. These studies suggested that targeting the BET proteins will benefit hyperinflammatory conditions associated with high levels of cytokine production [44]. In ovarian cancer, JQ1 suppresses tumor growth associated with cell cycle arrest, apoptosis induction, and metabolic alterations [45]. In Ewing Sarcoma, co-immunoprecipitation revealed an interaction of BRD4 with CDK9. Combined treatment of Ewing Sarcoma with BRD- and CDK9-inhibitors resulted in enhanced responses compared to individual drugs not only in vitro but also in a preclinical mouse model in vivo [46]. A recent study reported that BETi GS-626510 (10 mg/kg twice a day treatment for 13 days) significantly decreased the in vivo uLMS growth compared to vehicle control [47]. In addition to the Bromo- and extra-terminal domain (BET) family, the role of the non-BET family has been investigated recently. For instance, non-BET family inhibitor NVS-CECR2-1 inhibits chromatin binding of CECR2 BRD and displaces CECR2 from chromatin within cells. NVS-CECR2-1 exhibits cytotoxic activity against various human cancer cells, killing SW48 colon cancer cells by inducing apoptosis [48]. In melanoma, the iBRD9 (TP-472) blocked tumor growth by suppressing ECM-mediated oncogenic signaling and inducing apoptosis [34]. In renal clear cell carcinoma, the combined analysis of BRD9 and other chromatin regulated genes showed a significant association with the high-risk groups and lower overall survival, providing a survival prediction model for further research investigating the role of the expression of BRD genes in cancers [49].

uLMS is a rare but extremely aggressive tumor characterized by therapy resistance. Consequently, uLMS patients commonly present high rates of tumor recurrence, progression and metastasis [4]. While the malignant potential of uterine fibroids is extremely low, the possibility of transforming of UFs to uLMS has been proposed [6,50]. Therefore, other mechanisms should play a critical role in the development and progression of this aggressive cancer [5,51]. In this study, we demonstrated for the first time that expression levels of non-BET family member BRD9 are aberrantly upregulated in uLMS compared to adjacent myometrial tissues and higher in uLMS cells as compared to myometrial and benign UF cells, respectively, indicating the key role of BRD9 protein in the pathogenesis and progression of this cancer.

The development and use of small chemical inhibitors are fundamental and critical to the preclinical evaluation of BRDs as targets. Recently, a novel non-BET inhibitor, TP-472, has been developed with high potency for BRD9 [52]. In this study, we investigated the impact of TP-472 on uLMS cells aberrantly overexpressing BRD9 and demonstrated that TP-472 significantly inhibited uLMS proliferation concomitantly with a dose dependent decrease in BCL-2 expression. This study is consistent with the previous observation that iBRDs induced apoptosis by activating the expression of several pro-apoptotic genes in melanoma and MLL-fusion leukemia cells [34,42].

To further determine the mechanistic action of BRD9 inhibition, we performed comparative transcriptome-wide RNA-sequencing in uLMS cells treated with vehicle or TP-472. Previously, transcriptome-wide mRNA profiling in melanoma cells demonstrated TP-472 not only upregulated pro-apoptotic genes such as BAX, CDKN1A, GADD45A, GAD45B, among others, associated with the p53 pathway, but also downregulated several extracellular matrix proteins, which are required for tumor growth [34]. Our transcriptomic profiling analysis in uLMS revealed that multiple pathways were altered in response to TP-472 treatment. For instance, signatures enriched in uLMS cells, including MYC targets, interferon-alpha/gamma, K-ras, TNF signaling via NFkB, and MTORC1 signaling were observed between TP-472 and the control group. These newly identified pathways in uLMS in response to TP-472 treatment suggest that TP-472 targets common pathways and oncogenic transcriptome networks based on different types of cancer cells. Notably, using the PGCNA approach, we identified nine modules affected by iBRD9. In this study, we focused on cell cycle and apoptosis modules that correlated with uLMS phenotype changes induced by iBRD9. The expression of genes belonging to these two models was significantly altered. In addition, we validated the expression of cell cycle and apoptosis-related genes and further demonstrated that TP-472 treatment increased the expression levels of the cell cycle arrest- and apoptosis-related genes, suggesting that TP-472 suppressed the uLMS growth via induction of both apoptosis and cell cycle arrest. Several Hub DEGs, including MYC, FOS, JUN, EGR1, SHH, GRIN1, SOX9, HRAS, IMP3, CDK1, TOP2A, TOP2B, among others, are identified upon iBRD9 treatment. MYC is known to cooperate with KRAS in driving many cancers and contributes to many cancer hallmarks [53]. The MYC gene is a proto-oncogene and encodes a nuclear phosphoprotein that plays a role in cell cycle progression, apoptosis, and cellular transformation. The expression changes of MYC as identified in the Hub DEGs are consistent with Hallmark pathway analysis, showing the MYC pathway is enriched in response to TP-472 treatment, indicating the important role of the MYC hub and related network in the pathogenesis of uLMS. This study observed the compensatory induction of MYC RNA expression and HRAS by iBRD9. Since MYC is known to undergo a variety of posttranscriptional modifications, including acetylation, ubiquitinoylation, phosphorylation, glycosylation, SUMOylation, and phosphorylation-directed isomerization, which influence its stability, location, and activity [54,55,56,57], increased effort to deciphering the role of MYC pathway and it network in the pathogenesis of uLMS studies is needed. Importantly, simultaneously inhibiting BRD9 and MYC may efficiently induce growth arrest and apoptosis of uLMS cells, suggesting the potential of a combination treatment strategy.

The jun and fos families are involved in the regulation of cell proliferation, differentiation, and transformation [58,59]. Fos can dimerize with proteins of the jun family, thereby forming the transcription factor complex AP1 and enhancing the transcriptional activity. The AP1 complex can interact with bZIP proteins and structurally unrelated transcription factors. This cooperative recruitment of transcription factors, coactivators and chromatin remodeling factors to promoter and enhancer regions mediates the selective regulation of transcription activity [60]. In this study, the fos and jun expression was increased in iBRD9-treated uLMS cells. Previous studies demonstrated a discrepancy between jun/fos proto-oncogene mRNA and protein expression in other diseases [61,62]. Further functional studies are needed to investigate the role of fos/jun families in response to iBRD9 treatment. EGR1 as an additional transcriptional regulator in Hub DEGs plays an important role in regulating the transformation, cell survival, proliferation, and cell death. In this study, TP472 increased the expression of EGR1 in uLMS, providing a mechanism of TP-472-induced inhibition of cell proliferation. Notably, the role of EGR1 in tumor formation is controversial in diseases and conditions. For example, EGR1 functions as a tumor suppressor by monitoring DNA damage, promoting cell apoptosis, and enhancing the anticancer effects of radiotherapy and chemotherapy. EGR1 can activate the expression of p53/TP53, and thereby helps prevent tumor formation. However, in certain circumstance such as hypoxic microenvironments, the increase in EGR1 expression maintains tumor cell survival, proliferation, metastasis, and tumor angiogenesis [63]. SOX9 (hub DEG) as a transcriptional factor is also identified in this study.

Several transcription factors of BRD9 have recently been identified [64]. Notably, the master transcription factor Sox 17 recruits BRD to a subset of enhancers associated with genes involved in the cell cycle and angiogenesis, among others. In this regard, BRD9 acts as a molecular bridge between the subset of enhancers and the transcription machinery [64]. Therefore, a deep dive into the role and functional mechanism of BRD9 transcription factors will help us to understand the pathogenesis of malignant uLMS.

Notably, the SHH pathway is observed in our study as the Hub pathway in response to TP-472 treatment. We have demonstrated that the Hedgehog pathway played a critical role in uLMS pathogenesis via GLI molecules [5,65,66]. In this study, we demonstrated that inhibition of BRD9 downregulated the expression of GLI1/2, the key components in the Hedgehog pathway, indicating the interplay between BRD9 and the Hedgehog pathway implicated in the uLMS pathogenesis. HRAS plays an important role in cell division, the process by which cells mature to carry out specific functions (cell differentiation), and the self-destruction of cells (apoptosis) [67]. It has been shown that TP-472 treatment leads to upregulation of GADD45A, the pro-apoptosis gene in melanoma cells [34]. GADD45A has a direct role in p38 activation by HRAS. Another required component for HRAS growth arrest is GADD45A, which is known to be regulated by p53 [67]. Interestingly, the expression level of HRAS was increased in TP-472-treated uLMS cells suggesting that HRAS might cause apoptosis in TP-472-treated uLMS cells via GADD45A.

It has been reported that pathways involved in the regulation of cell cycle and apoptosis were most significantly enriched in direct targets of IMP3 at transcriptome and translatome levels, respectively. IMP3 silencing downregulated several well-known apoptosis regulators such as BIRC5, RAF1, and ESPL1 [68]. Overexpression of IMP3 in TP-472-treated uLMS cells suggests that IMP3 plays a significant role in the uLMS pathogenesis.

The role of CDK1 in gynecological cancer is limited to ovarian and endometrial cancer [69,70]. The inhibition of CDK1 activity induced cell apoptosis and caused the G2/M phase arrest of cell cycle in endometrial cancer cells [70]. Zhang et al. showed that CDK1 inhibition by shRNA repressed the cell proliferation, and the cell numbers in G2/M phase and cell apoptosis rate were increased in both SK-OV-3 and OVCAR-3 cells [69]. Ying et al. found that CDK1 was one of the hub genes in the pathogenesis of endometrial cancer. CDK1 inhibitor, RO3306, induced cell apoptosis and caused G2/M phase arrest of the cell cycle in endometrial cancer cells [70]. Accordingly, our bioinformatics analysis results revealed that the expression level of CDK1 in TP-472-treated uLMS cells was decreased. Further experimental studies are required to determine whether CDK1 could serve as a novel therapeutic target for uLMS patients.

TOP2A is expressed predominantly in cycling cells and plays key roles in DNA replication, chromosome segregation, and transcription, while TOP2B participates mainly in transcription, and it is ubiquitously expressed in both cycling and postmitotic cells [71]. If sister chromatids are not fully separated, cells will be arrested at the G2 phase. Accordingly, by blocking the TOP2A’s function after chromosome condensation, cells arrested at metaphase, chromosomes failed to separate, and anaphase bridges formed, resulting in partial or complete chromosome gains or losses and polyploidy [72]. Histone deacetylases (HDACs) modify nucleosomal histones. The results of the coimmunoprecipitation experiments indicated that TOP2A and TOP2B are substrates for HDAC1 and HDAC2, Class I HDAC enzymes, and that complexes containing HDAC1 or HDAC2 can increase TOP2 activity [73]. Our previous study showed that HDAC inhibitors induced apoptosis of uterine fibroid cells and cell cycle arrest [74]. In this study, we demonstrated that inhibition of BRD9 downregulated the expression of TOP2A and TOP2B; therefore, it is plausible that iBRD9 exerts its effect via HDACs. These results suggested that iBRD9 altered gene set comprises a distinct and functionally connected group of targets in uLMS phenotype. We will perform further analysis with other enriched modules to obtain more comprehensive knowledge of iBRD9 action and mechanism. These hub genes have great predictive value for the prognosis and progression of uLMS and may contribute to the understanding of basic and clinical research on uLMS.

Previously, it has been reported that crosstalk between different epigenetic mechanisms regulated gene expression. To determine the relation between BRD9 and other histone marks, we analyzed the epigenome alterations associated with differentially expressed genes (DEGs) upon TP-472 treatment. Notably, we observed that DEGs in response to TP-472 treatment were significantly associated with the enrichment of several histone marks, including H3K27me3, H3K4me1, H3K4me2, and H3K4me3. It was previously reported that BET BRDs play a role in the installing histone methylation. For instance, blocking readers of H3K27ac by BET inhibitor (JQ1) abolished H3K27ac-induced H3K4me3 installation and downstream gene activation [75]. Although epigenetic changes correlating to the uLMS phenotypes have been reported in uLMS [65,76,77,78], our findings herein, to our knowledge, present the first evidence showing that non-BET BRDs may play a critical role in crosstalk with histone marks, implicating non-BET BRDs in the complex oncogenic epigenome network contributing to the pathogenesis of malignant uLMS.

miRNAs are short (~22 nucleotides) non-coding RNAs that regulate post-transcriptional gene expression by influencing the translation or stability of target mRNAs. The interplay between miRNAs and epigenomic marks has been widely reported, including uLMS [79,80]. In this study, we demonstrated that miRNAs might play a critical role in regulating gene expression in uLMS cells in response to TP-472 treatment. We identified several miRNAs that correlated with DEGs either up or downregulated by TP-472 treatment. These miRNAs have not been reported in the uLMS, broadening the knowledge that BRDs may alter the transcriptome via miRNA-mediated gene regulation. Our studies emphasize that pharmacological inhibition of BRD9 suppressed the development of uLMS. Additional studies are needed to uncover the BRD9 mechanism of action further.

We propose a mechanistic model for targeting non-BET BRDs in uLMS based on our novel findings herein that: (1) BRD9 expression is abnormally upregulated in uLMS tissues and cells; (2) targeting BRD9 alters the uLMS phenotype with a decrease in cell proliferation and anti-apoptotic marker BCL-2; (3) TP-472 altered several key pathways and reprogrammed the oncogenic epigenome and miRNA network to suppress the uLMS phenotype, leading it to be a new option for uLMS treatment (Figure 9B).

## 5. Conclusions

Our study demonstrates for the first time that BRD9 expression is deregulated in human uLMS tissues and cell lines compared to MM^+uLMS^ and uterine fibroid/myometrial cell lines, respectively. Furthermore, inhibition of BRD9 suppresses the uLMS phenotype by sculpting the transcriptome, reprogramming the oncogenic epigenome, and altering the miRNA-mediated gene regulatory network (Figure 9B). Our studies broaden the knowledge about the role of BRD proteins in the pathogenesis of cancers. Accordingly, targeting non-BET BRDs in malignant uLMS may provide a promising and novel strategy for treating patients with this aggressive uterine cancer.

## Figures and Tables

**Table 1:** Characteristics of uLMS samples.

Case Number	Type of LMS	Age at Diagnosis	Tumor Size (cm)	Stage	Recurrence	Necrosis	Metastasis	Survival Status
1	Conventional	58	7	IB	N	focal coagulative necrosis	None	A
2	Conventional	58	8	I	Y	extensive hyaline necrosis. focal coagulative necrosis	Lung	D
3	Conventional	42	7.1	IB	N	coagulative tumor cell necrosis and hyaline necrosis	Brain and lung	A
4	Conventional	62	7.9	IB	Y	focal coagulative necrosis	Lung	A
5	non-conventional	55	17	IIIC1	N	focal coagulative necrosis	Ovary & Lung	D
6	non-conventional	54	18.8	IIIB	Y	focal coagulative necrosis	Abdominal wall + Bowel	D
7	Conventional	54	27	II	Y	focal coagulative necrosis	Omentum	A
8	Conventional	61	9.5	IIIB	N	focal coagulative necrosis	Omentum, Small intestine, Liver	A
9	Conventional	42	24.6	IB	N	focal coagulative necrosis	None	D

**Table 2:** Antibodies used in the study.

Antibodies	Company	Catalog Number	Source	Application	Dilution
BRD9	Cell Signaling	58906	Rabbit	WB	1:1000
Bcl2	Abcam	ab182858	Rabbit	WB	1:1000
β-actin	Sigma	A5316	Mouse	WB	1:8000
BRD9	Abcam	ab259839	Rabbit	IHC-P	1:1000
SMA	Dako	M0851	Mouse	IHC-P	1:1600
Desmin	Santa Cruz	SC-14026	Rabbit	IHC-P	1:800
HMB45	Dako	M0634	Mouse	IHC-P	1:100

**Table 3:** Primer information in this study.

Gene Symbol	Primer Sequences	F or R	Assay	Species	Size (bp)	Accession
CDKN1A	CGGAACAAGGAGTCAGACATT	F	q-PCR	Human	105	NM_00389.5
CDKN1A	AGTGCCAGGAAAGACAACTAC	R	q-PCR	Human	105	NM_00389.5
BAK	AGGGCTTAGGACTTGGTTTG	F	q-PCR	Human	100	U16811.1
BAK	GGGATTCCTAGTGGTGTTGATA	R	q-PCR	Human	100	U16811.1
18 S	CACGGACAGGATTGACAGATT	F	q-PCR	Human	119	NR_145820
18 S	GCCAGAGTCTCGTTCGTTATC	R	q-PCR	Human	119	NR_145820

**Table 4:** The significant terms of the modules based on the 9 different enrichment databases.

Module	Mid Night Blue	Yellow	Cyan	Dark Green
GO_Biological_Process_2021	Regulation of mitochondrial outer membrane permeabilization involved in apoptotic signaling pathway (GO:1901028) (1.38 × 10^−5^)	Apoptotic process (GO:0006915) (2.00 × 10^−5^)	Translational termination (GO:0006415) (8.54 × 10^−31^)	Mitotic cell cycle phase transition (GO:0044772) (3.36 × 10^−7^)
MSigDB_Hallmark_2020	TNF-alpha Signaling via NF-kB (0.000138704)	TNF-alpha Signaling via NF-kB (1.08 × 10^−16^)	Myc Targets V1 (1.25 × 10^−39^)	PI3K/AKT/mTOR Signaling (0.031214077)
WikiPathway_2021	MAPK Signaling Pathway WP382 (0.001957412)	Sudden Infant Death Syndrome (SIDS) Susceptibility Pathways WP706 (0.000455634)	G1 to S cell cycle control WP45 (0.037948453)	Cell cycle WP179 (1.90 × 10^−5^)
Reactome_2015	TRAF6-mediated induction of NFkB and MAP kinases upon TLR7/8 or 9 activation Homo sapiens R-HSA-975138 (0.093688577)	MAPK family signaling cascades Homo sapiens R-HSA-5683057 (0.019875184)	Cyclin E associated events during G1/S transition Homo sapiens R-HSA-69202 (9.09 × 10^−10^)	Cell Cycle Homo sapiens R-HSA-1640170 (3.50 × 10^−11^)
ENCODE_Histone_Modifications_2015	H3K4me1 (2.98 × 10^−15^)	H3K27me3 (4.96 × 10^−48^)	H3K4me3 (5.19 × 10^−10^)	H3K4me3 (1.91 × 10^−6^)
TargetScan_microRNA_2017	hsa-miR-4776-5p MicroRNAs (0.001954789)	hsa-miR-4727-3p (0.001242581)	hsa-miR-1284 (0.021500132)	hsa-miR-3682-3p MicroRNAs (5.49 × 10^−14^)
InterPro_Domains_2019	NFkappaB IPT domain (0.007433747)	Death domain (0.001693697)	LSM domain (1.28 × 10^−9^)	Protein kinase domain (4.49 × 10^−5^)
Pfam_Domains_2019	Pkinase (0.000212328)	Death (0.000791274)	LSM (5.50 × 10^−10^)	Pkinase (3.62 × 10^−5^)
Jensen_COMPARTMENTS	Neuron part (5.89 × 10^−7^)	BCL-2 complex (3.34 × 10^−8^)	Intracellular ribonucleoprotein complex (5.21 × 10^−49^)	Cellular component (5.48 × 10^−22^)

## Data Availability

Raw FASTQ files were deposited in the NCBI Gene Expression Omnibus (GSE205775).

## Supplementary Materials

The following supporting information can be downloaded at: https://www.mdpi.com/article/10.3390/cells11142160/s1, Figure S1: Validation of *p21* and *BAK* gene expression; Table S1: Top 40 hub genes shared between DEGs and four selected modules.
