# Supplementary material for: The Functional Role and Regulatory Mechanism of Bromodomain-Containing Protein 9 in Human Uterine Leiomyosarcoma
Source: Cells. 2022 Jul 10;11(14):2160. doi: 10.3390/cells11142160 (PMC9323884; doi:10.3390/cells11142160)
Supplement: Supplementary file 1 [file cells-11-02160-s001.zip › cells-1783020-supplementary.pdf]

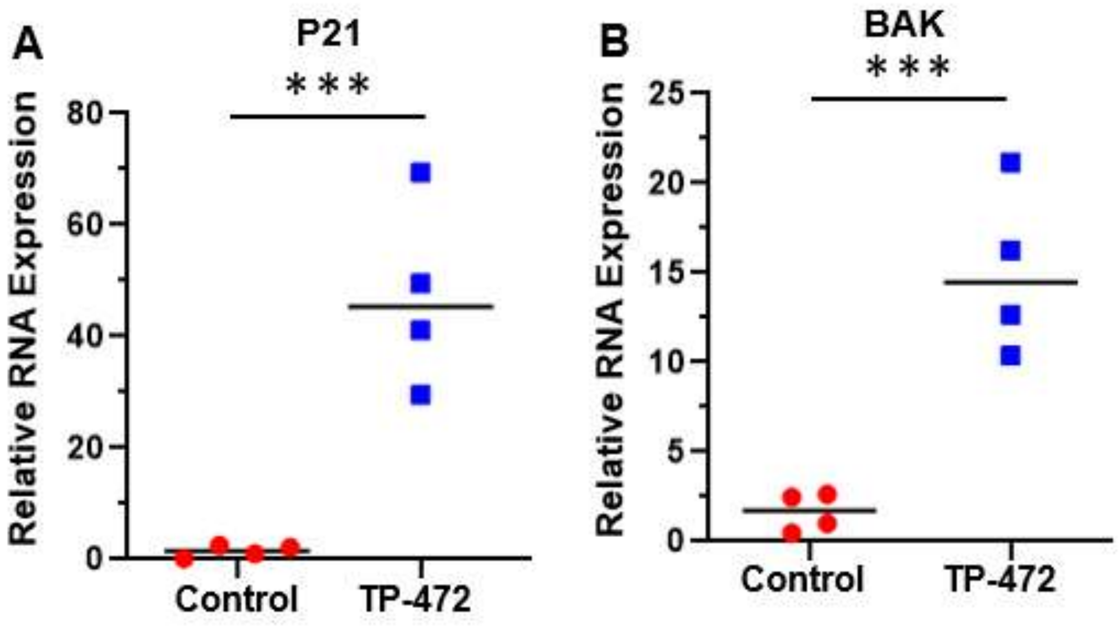

Figure S1. Validation of *p21* and *BAK* gene expression

Table S1. Top 40 hub genes shared between DEGs and four selected modules

Genes	Modules	FC	Degree	Genes	Modules	FC	Degree
MYC	Midnight Blue	1.56	165	CCT2	Cyan	1.61	160
FOS	Midnight Blue	1.50	81	NOP56	Cyan	1.64	154
SHH	Midnight Blue	4.66	70	NOP58	Cyan	1.74	149
GRIN1	Midnight Blue	2.54	56	NIP7	Cyan	1.92	141
SOX9	Midnight Blue	2.02	55	PA2G4	Cyan	1.55	139
TERT	Midnight Blue	1.68	47	HSPA8	Cyan	1.75	139
STX1A	Midnight Blue	1.78	45	IMP3	Cyan	1.62	136
CEBPB	Midnight Blue	1.75	44	EIF2S1	Cyan	1.52	124
EGR1	Midnight Blue	1.63	42	RPL26L1	Cyan	1.58	118
GATA2	Midnight Blue	2.26	39	CYCS	Cyan	1.86	117
JUN	Yellow	3.13	93	CDK1	Dark Green	-1.56	91
FN1	Yellow	2.40	87	TOP2A	Dark Green	-1.52	71
HRAS	Yellow	1.60	80	CHEK2	Dark Green	-1.69	61
CCND1	Yellow	2.21	68	WDHD1	Dark Green	-1.52	56
MRTO4	Yellow	2.16	53	TOP2B	Dark Green	-1.52	39
KIT	Yellow	20.37	53	STAG2	Dark Green	-1.51	34
BYSL	Yellow	1.75	49	MIS18BP1	Dark Green	-1.53	28
RRP9	Yellow	2.71	49	HLTF	Dark Green	-1.78	25
PDCD11	Yellow	1.69	49	FGF2	Dark Green	-1.56	25
LEF1	Yellow	6.22	49	TIA1	Dark Green	-1.60	17
